# Platelet protein biomarker panel for ovarian cancer diagnosis

**DOI:** 10.1186/s40364-018-0118-y

**Published:** 2018-01-12

**Authors:** Marta Lomnytska, Rui Pinto, Susanne Becker, Ulla Engström, Sonja Gustafsson, Christina Björklund, Markus Templin, Jan Bergstrand, Lei Xu, Jerker Widengren, Elisabeth Epstein, Bo Franzén, Gert Auer

**Affiliations:** 1 Department of Obstetrics and Gynaecology, Academical Uppsala University Hospital, Uppsala University, SE-751 85 Uppsala, Sweden; 2 Institute of Women’s and Children’s Health, Karolinska Institute, SE-171 76 Stockholm, Sweden; 3 Department of Oncology and Pathology, Cancer Centre Karolinska, Karolinska Institute, SE-171 76 Stockholm, Sweden; 4 Department of Epidemiology and Biostatistics, MRC-PHE Centre for Environment and Health, School of Public Health, Imperial College London, St. Mary’s Campus, Norfolk Place, W2 1PG, London, England UK; 5 Ludwig Institute for Cancer Research Ltd, Box 595, SE-751 24 Uppsala, Sweden; 6 NeoProteomics AB, Cancer Centre Karolinska, SE-17176 Stockholm, Sweden; 7 NMI Natural and Medical Sciences Institute at the University of Tübingen, 72770 Reutlingen, Germany; 8 Experimental Biomolecular Physics, Department of Applied Physics, Royal Institute of Technology, AlbaNova University Center, SE-106 91 Stockholm, Sweden; 9 Department of Obstetrics and Gynaecology, Department of Clinical Science and Education, Södersjukhuset, SE-118 83 Stockholm, Sweden

**Keywords:** Ovarian cancer, Platelet proteome, Biomarker, Liquid biopsy

## Abstract

**Background:**

Platelets support cancer growth and spread making platelet proteins candidates in the search for biomarkers.

**Methods:**

Two-dimensional (2D) gel electrophoresis, Partial Least Squares Discriminant Analysis (PLS-DA), Western blot, DigiWest.

**Results:**

PLS-DA of platelet protein expression in 2D gels suggested differences between the International Federation of Gynaecology and Obstetrics (FIGO) stages III-IV of ovarian cancer, compared to benign adnexal lesions with a sensitivity of 96% and a specificity of 88%. A PLS-DA-based model correctly predicted 7 out of 8 cases of FIGO stages I-II of ovarian cancer after verification by western blot. Receiver-operator curve (ROC) analysis indicated a sensitivity of 83% and specificity of 76% at cut-off >0.5 (area under the curve (AUC) = 0.831, *p* < 0.0001) for detecting these cases. Validation on an independent set of samples by DigiWest with PLS-DA differentiated benign adnexal lesions and ovarian cancer, FIGO stages III-IV, with a sensitivity of 70% and a specificity of 83%.

**Conclusion:**

We identified a group of platelet protein biomarker candidates that can quantify the differential expression between ovarian cancer cases as compared to benign adnexal lesions.

**Electronic supplementary material:**

The online version of this article (10.1186/s40364-018-0118-y) contains supplementary material, which is available to authorized users.

## Background

Epithelial ovarian cancer is characterised by an asymptomatic growth in the abdominal cavity. In 75% of all cases, it is only detected at an advanced stage. The 5-year survival rate in FIGO stages I-II is over 90%, compared to around 30% in stages III-IV [1, 2]. The sensitivity of the CA-125 tumor marker for the detection of non-advanced epithelial ovarian cancer ranges from 50% to 70%, and this parameter alone is not recommended for differentiating between a benign and a malignant adnexal mass [3]. An expertly conducted transvaginal sonography (TVS) is a primary method for evaluation of ovarian and pelvic tumors, as it is able to discriminate between benign and malignant conditions with 90% sensitivity and 94% specificity using the International Ovarian Tumor Analysis (IOTA) pattern recognition [4, 5]. Despite high performance of TVS, the assessment will be inconclusive in around 8% of cases, also in the hands of an expert examiner [4]. Commonly used tumor markers, such as CA-125, HE4 and the Risk of Malignancy Index (RMI), did not improve, but rather deteriorated assessment in these difficult to classify tumors [6].

A several-fold increase in a patient’s platelet count is a common observation in cancer. A high preoperative platelet count is associated with early relapse in non-advanced epithelial ovarian cancer [7] and colorectal cancer [8]. Platelets influence angiogenic and immunological processes in cancer [9, 10], as well as directly protect tumor cells [7]. Proteomic analysis of platelets has identified several potential cancer markers [11]. Among them are angiogenic factors that have been shown to be sequestered by platelets [12] and delivered to the site of activated endothelium within an early tumor [11]. Detection of platelet-derived growth factor, platelet factor 4 (PF-4) and platelet-derived endothelial cell growth factor was suggested for diagnosis of several cancers [13]. Upon platelet activation, PF-4, vascular endothelial growth factor (VEGF) and fibrinogen undergo significant spatial rearrangements, detectable only by super resolution stimulated emission depletion (STED) microscopy [14].

The proteome of platelets in ovarian cancer has not been previously studied. The hypothesis of the current study is that knowledge of quantitative alterations of proteins in platelets can become the basis for non-invasive diagnosis of ovarian cancer and evaluation of the malignant potential of adnexal lesions.

## Methods

The study comprised three phases:

1. Platelet pellets were prospectively collected from patients with benign adnexal lesions and ovarian cancer. This clinical material was subjected to two-dimensional (2D) gel electrophoresis, statistical analysis of protein expression, and subsequently, mass spectrometry-aided identification of protein biomarker candidates.
2. Antibody identification and confirmation of protein identification by western blot.
3. Verification of our biomarker candidate protein panel using western blot and DigiWest, and evaluation of sensitivity and specificity for detecting ovarian cancer.

### Clinical material

Blood samples were obtained from volunteering women with adnexal lesions and suspected ovarian cancer. Approval for the study was given by the local ethical committee of Stockholm County Council, Dnr 2010/504–31. Cases were coded as “TR” followed by a number, and the coding was saved separately from the personal information of patients. Clinical material from 114 patients was prospectively collected between 2011 and 2014 at the Department of Obstetrics and Gynaecology, Karolinska University Hospital-Solna, Stockholm, Sweden (Table 1, Additional file 1: Table S1). Peripheral venous blood was drawn from the antecubital vein of each subject. The requirement for inclusion in the study was collection of a blood sample prior to any invasive diagnostic or treatment procedures. Patients with another known active cancer were excluded from the study. Patient records included age at diagnosis, co-morbidities, medication with coagulation and platelet aggregation blockers, optionally - TVS according to IOTA criteria, and post-operative histopathological conclusion. Randomization of the material was performed prior to experimental procedures.

**Table 1:** Description of the clinical material

Diagnosis and International classification of disease (ICD) coding					
Benign lesions		Epithelial ovarian cancer, C56.9			
	n	stages I-II	n	stages III-IV	n
Serous ovarian cyst, N82.3	19	serous	2	serous	47
Ovarian fibrom, N82.3	10	mucinous	1	endometrioid	1
Dermoid ovarian cyst, N82.3	5	endometrioid	2	clear-cell	1
Endometriosis cyst, N80.1	8	clear-cell	3		
Mucinous ovarian cyst, N83.2	10				
Non-cancer ascites, R18.9	1				
Paratubar cyst, Q50.5	2				
Uterine myom, D25.9	2				
Total	57		8		49
Transvaginal sonography, IOTA classification					
Ultrasound assessment	Certainty in the assessment			Histopathology	n
Benign	certainly benign			benign	7
Benign	probably benign			benign	16
Benign	uncertain			benign	6
Borderline tumor	uncertain			benign	4
Malignant	probably malignant			benign	2
Malignant	certainly malignant			malignant	1^a^
Malignant	certainly malignant			malignant	13^b^
no IOTA-based examination				benign	22
no IOTA-based examination				malignant	7^a^
no IOTA-based examination				malignant	36^b^
Total					114
		Medication:			
Comorbidities	n	coagulation/aggregation blockers			n
None	99		none		111
One or few following diseases:	15		Warfarin		1
Breast cancer remission	4		Dabigatran		1
Cardiovascular	14		Aspirin		1
Rheumatic	2		Total		114
Endocrine	4		Experimental setup, n		
Astma	2	Method/Statistics	Benign	Ovarian cancer, stage	
Hepatitis C	1		lesions	I-II	III-IV
Total	114	2D/PCA	28	8	32
		2D/PLS-DA	25	8	30
		Western blot/PLS-DA	20	8	20
		DigiWest/PLS-DA	29	0	30

^a^stages I-II

^b^stages III-IV

### Isolation of platelets from peripheral blood

Peripheral venous blood was drawn into 4.5 ml vacutainer plastic whole blood collection tubes with spray-coated K_2_EDTA (Vacutainer, BD, Franklin Lakes, NJ, USA) and processed within 30 min. Isolation of platelets was performed by three centrifugation steps. Exclusion of erythrocytes and leucocytes was achieved by centrifugation of whole blood at 1500 relative centrifugal force (RCF) for 10 min at +4 °C, and the platelet-rich plasma obtained was subjected to a second centrifugation at 3000 RCF for 10 min at +4 °C [15]. The resulting platelet pellet was re-suspended in 500 μL 0.9% NaCl and centrifuged at 3000 RCF for 10 min at +6 °C. The quality and purity of the platelet isolation was confirmed by light microscopy after immunostaining with an antibody against CD61 (119,992, Abcam, Cambridge, UK). In addition, fluorescence-activated cell sorting (FACS) of selected cases using the antibody against p-selectin, or CD62 (348,107, BD Biosciences-Europe) was also used to assess quality and purity. Platelet pellets were aliquoted to avoid excess freeze-thaw cycles and stored at −70 °C.

### Two-dimensional gel electrophoresis

The platelet fractions for 2D gel electrophoresis were lyophilized and resuspended in lysis buffer; the protein concentration was determined using the Bradford protein analysis protocol [16] and the Versa Max Microplate reader (Molecular Devices, Sunnyvale CA, USA). Samples of 75.0 μg protein were subjected to 2D gel electrophoresis as previously described [17]. One 2D gel per clinical case was included into analysis after evaluation of the gel quality, i.e., absence of protein degradation protein spot smearing or overstraining. The expression level of protein spots in 2D gels was analysed using Progenesis SameSpot software (Nonlinear Dynamics, London, UK). The cut-off for selection of protein spots was a relative expression difference of 1.5-fold, *p* < 0.05, power > 0.8, *q* < 0.05, as evaluated by analysis of variance (ANOVA) by the SameSpot software.

### Search parameters and acceptance criteria for MS/MS and peptide mass fingerprint (PMF)

Protein spots selected for identification were excised from the gels, treated for in-gel digestion, and subjected to MALDI TOF mass spectrometry carried out on the Ultraflex III TOF/TOF (Bruker Daltonics, Bremen, Germany). Peptide spectra were internally calibrated using trypsin autolytic peptides. A peak list generating software, Data Analysis 3.2 (Bruker Daltonics, Bremen, Germany), was used. In selected cases, MS/MS was performed with acceptance score exceeding 30. Mass tolerance for fragment ions was 0.5 Da. Based on the obtained peptide spectra, identification of the proteins was performed using the MASCOT (Matrix Science, London, England) and the “NCBInr” database. The deviation of mass did not exceed 0.05 Da. Probability of identification was evaluated according to score value, sequence coverage, and matched peptides. All other steps were performed as previously described [17].

### Western blot analysis

A semi-quantitative dual label fluorescent detection western blot analysis was performed using the same patient cases that were subjected to 2D gel electrophoresis (Table 1). Samples were diluted in 4× lithium dodecyl sulfate (LDS) buffer and 10× reducing agent to a concentration of 1 μg/μl, and then incubated for 10 min at 70 °C. Four NuPAGE 4–12% Bis-Tris Gel, 1.0 mm × 15 wells were run in parallel; a total of 10 μg protein per case was loaded. Gel running conditions were as follows: 200 V for 60 min in XCell SureLock Mini-Cell EI0001 (Life Technologies, Stockholm, Sweden), followed by incubation of gels in transfer buffer containing 10% methanol and blotting to a nitrocellulose membrane for 1 h at 30 V (BioRad Power Pac 100 and Hoefer EPS 2A200). Membranes were incubated with Odyssey blocking buffer (Li-Cor Biosciences, East Chesterton Ward, UK) with addition of 0.1% Tween 20 for one hour, and then with the combination of commercially available primary antibodies, GAPDH and anti-14-3-3-gamma for loading controls [18] (Additional file 1: Table S2) with rotation at +4 °C overnight. After washing with PBS, each membrane was incubated with secondary antibodies (IRDye 2nd Ab Goat anti-Rabbit 680, 1:10,000 and IRDye 2nd Ab Goat anti-Mouse 800, 1:15,000, Li-Cor Biosciences, East Chesterton Ward, UK) at room temperature for 1 h. After washing with PBS, each membrane was scanned using the Odyssey SA Infrared Imaging System (Li-Cor Biosciences, East Chesterton Ward, UK).

### DigiWest analysis

For each platelet lysate, two technical repeats (20 μg protein per lane) were subjected to SDS-PAGE using 4–12% Bis-Tris gradient gels. After blotting with primary antibodies (Additional file 1: Table S2) and biotinylation of the proteins, individual sample lanes were cut into 96 molecular weight fractions (0.5 mm each) and proteins were eluted. Eluted proteins from each molecular weight fraction were loaded onto color-coded neutravidin-coated Luminex bead sets (MagPlex, Luminex, Austin TX, USA), and the antibody specific signals were analysed (DigiWest analysis, version 3.8.5.2, Excel-based) [19].

### Multivariate methods (PCA, PLS-DA, OPLS-DA)

Principal components analysis (PCA) [20] is the most widely used (non-supervised) multivariate method, and the root to most others. By finding covariance between multiple (correlated) variables in a single dataset **X**, it sequentially defines (uncorrelated, or orthogonal) principal components, with decreasing amount of variance explained, built from weighted initial variables. A number of interesting components can be selected, as variation in the dataset is reframed as structured (e.g. biological information) or residual (noise). Each component defines a percentage of variation of the original dataset, and is described by two vectors: scores, representing the score of each sample in the newly created principal component; loadings, representing the weight of each initial variable in the principal component. PCA performs dimensionality reduction, thus allowing one to condense most of the information in a large dataset into a small number of components. Its scores and loadings can be plotted and used as an exploratory technique, to find relations between variables, detect groups and trends in the samples, as well as outliers.

Projection to latent structures (PLS) is a well established (supervised) method for the analysis of complex multivariate datasets, as found in the –omics fields, including proteomics [21, 22]. In originates from partial least squares regression, a multivariate analysis method which relates two matrices (**X** with the actual data or independent variables, and **Y/y** as responses or dependent variables) by maximizing the covariance of their latent variables. The 2-class PLS-DA is simply a particular type of PLS in which the dependent variable is a “dummy” binary (0/1) class **y**-vector. PLS-DA targets complementary objectives: as a discriminant method, it allows discrimination/prediction of class for test samples; as a multivariate method it shows the relationship among variables in the dataset through the creation of latent variables, built using weighted original variables; as a linear method, it allows one to visualize and understand which variables in the data are more relevant for the class discrimination.

Orthogonal PLS (OPLS) [23] is a modification of the PLS method, and while both models have the same model statistics and prediction capability, OPLS allows for easier interpretation of the relevant variables than PLS. The reason is because apart from dividing variation into systematic and residual (as PLS), OPLS also divides the systematic variation into predictive (related to the phenomenon in study) and orthogonal (structured variation related to other factors, such as age and gender). This property is advantageous when interpretation of the results, rather than prediction, is the main objective of the analysis. In the context of OPLS-DA the statistically significant predictive loadings show which initial variables are important in the discrimination of class, and in which class these variables have higher values.

### (O)PLS-DA number of latent variables and validation [24]

(O)PLS-DA models can separate data variability into systematic and random, and use the systematic one for building the actual model while discarding the random variation, or noise. In order to attain that objective, it is critical to select an appropriate number of latent variables for the model, which is achieved in general through the use sampling methods such as cross-validation (CV). In this strategy, one calculates multiple (O)PLS-DA models using subsets of the **X** data, while predicting the class (**y**) of the samples left out in each round. By doing this for models with different number of latent variables, one can evaluate how well predicted by the model are the samples that were left out in each of those models, and select the best one. It yields the following cross-validation’s statistics: R2X- fraction of the variance in **X** explained by each latent variable; R2- fraction of variance of **y** (class) explained by each latent variable; and most importantly Q2- the fraction of variance of **y** predicted by the model. A model with good modelling and predictive power is desired, and while R2X may be low (due to low number of variables in **X** explaining class discrimination), R2Y and Q2 should be the closest possible to each other, as well as close to a maximum of 1.

After a model is built, containing the appropriate number of latent variables, it undergoes appropriate validation procedures, notably cross-validation analysis of variance (CV-ANOVA), permutation test, and evaluation of cross-validation scores. CV-ANOVA compares the y predicted residuals of the model of interest with the variation around the global average using an F-test, resulting in a significance *p*-value for decisional purposes. Permutation test evaluates the statistical significance of estimated predictive power, by comparing R2 and Q2 of multiple models (where **y** was randomized) with R2 and Q2 of the actual model of interest. If the model is significant, its values are expected to be higher than for the y-randomized models. Cross-validation scores are scores that are calculated for each sample during cross validation, and can be visually or systematically evaluated to indicate over-fitting in case they differ from the regular scores.

### Prediction of test samples and variable relevance for class discrimination

After proper choice of number of latent variables and method validation, the model can be used for prediction of test samples, and to find out which variables are relevant for class discrimination.

As class membership is defined by the 0/1 dependent variable **y** vector in the training set, class membership of “new” samples in a test set is dependent on their predicted **y** value. Samples similar to class “1” are expected to yield values of **y** similar to 1, thus above a certain threshold, e.g. 0.5, while samples similar to the ones in class “0” will be predicted below that threshold.

To find out which variables are relevant for class discrimination, two methods are commonly used: the variable importance on the projection (VIP) method, which is an (unsigned) compact parameter to summarize the importance of each of the variables in PLS-type models with more than one latent variable; the other method can be used with OPLS-type models and checks if the confidence interval for the mean of the CV-calculated loadings crosses zero or not (in which case it is consistently positive or negative for each of the cross validation models).

PCA, PLS-DA and OPLS-DA were performed using SIMCA P v13.0 software (Umetrics AB).

### Experimental design and statistical rationale

Normalized expression values exported from SameSpot software of all protein spots (approximately 2000 spots in one 2D gel) were subjected to PCA analysis and PLS-DA.

PCA analysis was performed using the material from patients with benign adnexal lesions (28 cases), ovarian cancer, International Federation of Gynaecology and Obstetrics (FIGO) stages I-II (8 cases), and ovarian cancer, FIGO stages III-IV (32 cases) (Table 1). The PLS-DA model was based on the expression of all platelet protein spots in 2D gels in benign ovarian lesions (16 cases) and ovarian cancer, FIGO stages III-IV (20 cases). The predictive ability of the model was tested using 8 cases of the ovarian cancer, FIGO stages I-II, 9 cases of benign adnexal lesions and 10 cases of the ovarian cancer, FIGO stages III-IV. Protein spots selected by PLS-DA were ranked as variables of importance in the projection (VIP) by the strength of their input into the model. An analysis of the influence of co-morbidities and the intake of coagulation and platelet aggregation blockers was performed.

Western blot analysis for antibody identification and confirmation of protein identification was performed using 20 cases of benign adnexal lesions, 20 cases of ovarian cancer, FIGO stages III-IV and 8 cases of stages I-II (Table 1). The same cases were used for 2D electrophoresis (Additional file 1: Table S1). For protein panel verification, all analysed samples were randomized and distributed to four sets of gel separation batches. Each batch produced four equal gels, each of which, after transferring, was incubated with four different mixtures of primary antibodies against 16 proteins (Additional file 1: Table S2) for a total of 16 gels per data set. This experiment was repeated three times (sets 1–3). Protein expression levels were subjected to OPLS-DA, logistic regression and receiver-operator curve (ROC) analysis (MedCalc Software, Ostend, Belgium).

Verification using DigiWest analysis [19] was performed on an independent set of prospectively collected randomized platelet samples from patients with ovarian cancer, FIGO stages III-IV (*n* = 30), and benign adnexal control lesions (*n* = 29) (Table 1, Additional file 1: Table S1). Statistical analysis was OPLS-DA based.

## Results

### Cancer-related expression of platelet proteins in 2D gels

**Fig. 1:**
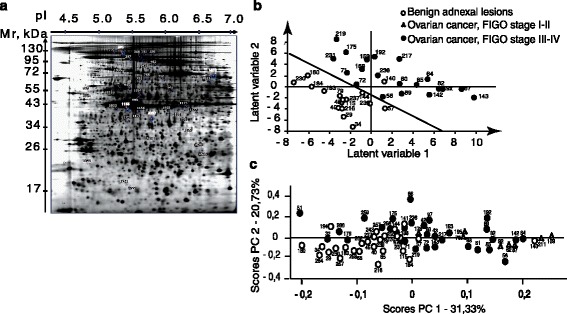
Proteomics-based analysis of platelet proteins was based on the separation of proteins according to mass (Mr, kDa) and charge (pI) by 2D gel electrophoresis with further analysis of the expression of protein spots for marker identification. **a** 2D gel electrophoresis diagram of platelet proteins. Circles and numbers indicate the identified biomarkers. **b** The PLS-DA-based cross-validated model based on the partial least squares discriminate analysis of 2D gels for benign adnexal lesions (white circle) and ovarian cancer, FIGO stage III-IV (black circle) in accordance to the expression of all protein spots in the gel. **c** Principal component analysis (PCA) showing separation of the generated 2D gels for benign adnexal lesions (white circle), ovarian cancer, FIGO stage I-II (triangle) and FIGO stage III-IV (black circle) in accordance to the expression of selected biomarkers; percentage of variance X explained by the two PCA components shown

Each 2D gel contained approximately 2000 protein spots (Fig. 1a). Initial PCA (Fig. 1c) of expression levels of significantly differentially expressed protein spots in 2D gels indicated a degree of separation between the three analysed patient groups: benign adnexal lesions, ovarian cancer, FIGO stages I-II and FIGO stages III-IV. PCA scores showing these groups are presented in Fig. 1c.

The PLS-DA cross-validated model based on the expression of all protein spots in 2D gels of benign adnexal lesions and ovarian cancer (FIGO stages III-IV) presented 96% sensitivity and 88% specificity for discrimination between these groups (Table 2a, Fig. 1b). The predictive ability of the built PLS-DA model was tested. We observed that all 8 cases ovarian cancer, FIGO stages I-II were recognised as cancer (1.00 sensitivity), and also 8 of 10 cases of ovarian cancer, FIGO stages III-IV (80%). Four out of 9 cases of benign adnexal lesions were recognised as benign (0.44 specificity) (Table 2a). Forty-three protein variables with VIP > 2 were selected for protein identification.

**Table 2:** Statistics for PLS-DA, OPLS-DA model, details for predictive and orthogonal parts; ROC analysis

A) PLS-DA-based analysis of protein spots expression in 2D									
Component	Latent variables	R2X (cum)	Q2 (cum)	CV-ANOVA, p-value	permutation test, p-value			Sensitivity	Specificity
Model	3	0.318	0.72	8,5 * 10–9	<0.001		calibration set	0.96	0.88
							validation set	1.00	0.44
B) OPLS-DA-based analysis of protein expression in western blot.									
Component	Latent variables	R2X (cum)	R2 (cum)	Q2 (cum)	CV-ANOVA, p-value	permutation test, *p*-value		Sensitivity	Specificity
Model	1 + 1	0.203	0.632	0.477	4.41E-14	<0.001	calibration set	0.83	0.89
Predictive	1	0.0717	0.632	0.477			validation set	0.88	not tested
Orthogonal	1	0.131	0						
C) ROC analysis of protein expression in western blot.									
Compared groups	AUC	standart deviation	95% confidence interval	z statistics	p-value			Sensitivity	Specificity
ROC 1	0.777	0.0418	0,695 to 0,859	6.639	<0,0001		ROC1	60	83.33
ROC 2	0.831	0.0501	0,733 to 0,930	6.615	<0,0001		ROC2	83.33	76.19
D) OPLS-DA-based analysis of protein expression in Digi west.									
Component	Latent variables	R2X (cum)	R2 (cum)	Q2 (cum)	CV-ANOVA, p-value	permutation test, p-value		Sensitivity	Specificity
Model	1 + 2	0.24	0.785	0.345	4.50E-03	<0.001	test set	0.7	0.83
Predictive	1	0.037	0.785	0.345					
Orthogonal	2	0.203							

*R2X* cumulative percentage of X variance explained, *R2* cumulative percentage of Y variance explained, *Q2* cumulative percentage of variance of Y predicted, *CV-ANOVA p-value* p-value of cross-validation ANOVA, *permutaion test p-value p*-value of (1000 iterations) permutation test

Co-morbidities and use of of coagulation and platelet aggregation blockers did not influence the predictive ability of the model. The PLS-DA model suggested a list of protein spots for protein identification as potential biomarkers, which could be evaluated from the VIP plot.

### Identification of platelet protein biomarkers and confirmation of identification

A total of 35 proteins were identified in 37 protein spots excised from 2D gels (Table 3).

**Table 3:** Protein identification

Protein spot number on 2D	Gene ontology name	Protein name	theoretical		experimental		NCBI accession number	Sequence coverage, %	Matched peptides	Total peptide count	score	msms
			pI	Mr, kDa	pI	Mr, kDa						
1	2	3	4	5	6	7	8	9	10	11	12	13
285	ACTN1	Alpha-actinin-1 isoform b	5.2	104	5.5	100	NP_001093	25	19	75	98	–
1836	ACTN4	Alpha actinin 4, partial	5.2	74	5.3	72	AAH15620	14	9	30	68	–
1153	ACTB	beta actin variant, partial	5.4	42	6.2	40	BAD96752.1	31	9	48	71	–
1803	ACTB	actin beta	5.2	39	5.3	42	BAG62914.1	30	10	54	81	–
1772	AP4A	Bis(5′-nucleosyl)- tetraphosphatase	5.1	17	5.4	17	NP_001152	27	5	36	52	1
1274	CAPZA1	capping actin protein of muscle Z-line alpha subunit 1	6	32	5.4	40	XP_005542394.1	54	11	60	106	–
911	CD41	Integrin alpha-IIb precursor variant	5.7	60	5.4	60	BAD92789.1	–	–	–	46	1
276	CD41	Integrin, alpha 2b (platelet glycoprotein IIb of IIb/IIIa complex, antigen CD41), isoform CRA_a	5.4	104	5.3	110	EAW51594.1	24	17	49	122	–
1761	CD42A	glycoprotein IX platelet	5.4	18	5.4	20	AAH30229	27	5	43	53	2
792	CD61	Platelet glycoprotein IIIa, partial	5	87	5.6	70	AAA52600	–	–	–	15	1
942	CNDP2	Cytosolic non-specific dipeptidase	5.6	53	6	55	BAG53426	22	8	48	51	3
1539	CRKL	Crk-lke protein	6.3	34	6.5	26	NP_005198	29	9	56	64	1
1525	ERP29	Erp28, endoplasmic reticulum resident protein 29 isoform 1 precursor	6.8	29	6.5	27	NP_006808	28	6	68	44	2
1854	FBG	Fibrinogen gamma chain	5.5	48	5.6	50	EAX04921	40	13	53	113	–
397	GELS	Gelsolin isoform a precursor (782 aa)	5.9	86	5.4	95	NP_000168	–	–	–	61	1
401	GELS	Gelsolin isoform b (731 aa)	5.6	81	5.7	90	NP_937895	12	9	50	45	2
311	HSPA9	Stress-70 potein, mitochondrial precursor	5.8	74	5.7	120	NP_001126860	27	17	66	100	3
1155	HP	Haptoglobin	6.3	38	5.4	42	AAI07588.1	25	10	61	71	–
482	HSPA8	Heat shock 70 kDa protein 1A/1B	5.4	70	5.7	80	P08107	13	6	56	29	–
1115	ILEU	Leukocyte elastase inhibitor	5.9	43	6.3	43	NP_109591	21	7	43	72	–
1177	MPST	3-mercaptopyruvate sulfurtransferase	6.8	35	6.4	37	BAD92061.1	33	8	39	75	–
1231	NSFL1C	NSFL1 cofactor p47 isoform X8	5	31	6.2	38	XP_011527607.1	25	5	46	38	1
1850	PGM1	phosphoglucomutase-1 isoform 1	6.3	62	6.8	70	NP_002624.2	11	5	32	33	–
1464	PHB	Prohibitin	5.5	28	5.7	30	XP_003912755.3	49	12	46	136	–
1054	RNH1	ribonuclease/angiogenin inhibitor 1	4.8	50	4.8	50	AAH11186.1	11	5	25	45	–
999	PRKAR1A	protein kinase cAMP-dependent type I regulatory subunit alpha	5.2	43	5.5	53	XP_004041145.1	35	13	72	102	
939	PRKAR2B	protein kinase cAMP-dependent type II regulatory subunit beta	5	45	4.8	55	BAG54705.1	19	6	27	41	2
1175	SRB6	Serpin B6	4.2	39	5.6	40	XP_011512978.1	34	10	45	76	–
1821	SRC	Protooncogene tyrosine-protein kinase Src	7.1	60	6.6	60	NP_005408.1	13	8	54	39	–
1801	TUBA4A	Tubulin alpha-4A chain	4.9	49	5.1	55	NP_001265481.1	23	10	56	73	–
1801	TUBA1C	tubulin alpha 1c	4.9	59	5.1	55	BAH11541.1	23	10	56	68	
758	TLN1	Talin 1	5.8	272	5.9	70	AAF27330	7	13	43	42	1
755	TLN1	Talin 1	5.8	272	5.8	70	AAF27330	9	15	46	54	–
292	TLN1	Talin 1	5.8	272	5.8	110	AAF27330	14	33	84	106	–
774	TLN1	Talin 1	5.8	272	6.4	70	AAF27330	–	–	–	25	1
1409	TLN1	Talin 1	5.8	272	6.4	34	AAF23322.1	6	15	39	47	–
1640	TPM1	Tropomyosin 1, alpha	5	20	4.7	20	XP_005254707	23	6	64	30	–
1894	TUBA6	Tubulin alpha-6 chain, partial	5	47	5.3	56	EHH66249.1	34	10	56	80	–
1894	TUBA8	tubulin alpha-8	5	43	5.3	56	XP_007469555.1	33	10	56	82	
1894	TUBA1C	tubulin alpha-1C chain	5	47	5.3	56	XP_004646666.1	35	10	56	81	
999	TUBB1	tubulin beta-1 chain isoform X7	5.1	43	5.5	53	XP_018872841.1	42	12	72	93	–
1850	WDR1	WD repeat-containing protein 1 iso 1	6.4	59	6.8	70	AAD05045	35	18	60	135	–

1 - number of a protein spot on 2-D gel,

2 - gene ontology name,

3 - protein name,

4 - isoelectric point of a protein spots according to the position on 2D gel,

5 - protein mass of a protein according to the position of a spot on 2D gel,

6 - isoelectric point of a protein spot as provided by Mascot search database,

7 - protein mass as provided by Mascot search database,

8 - protein accession number according to NCBI,

9 - matching of the experimental peptide sequence to a peptide sequence provided by NCBI,

10 - number of identified peptides that matched to the peptides of a peptide sequence provided by NCBI,

11 - total amount of peptides in a peptide sequence provided by NCBI,

12 - score provided by Mascot search engine,

13 - performed msms for the certainty of protein identification.

A number of proteins were excluded due to low quality and/or low expression level that made identification by mass spectrometry unfeasible. Visual representation of the location of the identified spots may be found in Fig. 1a.

**Fig. 2:**
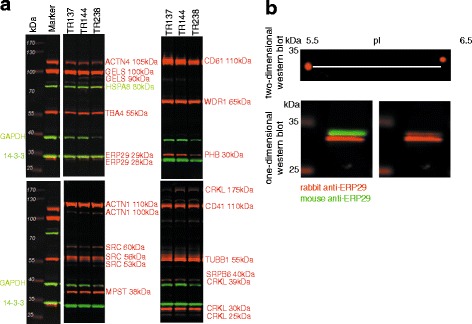
Western blot analysis. **a** Confirmation of protein identification by western blot using antibodies against 15 selected proteins, and normalisation against14–3-3-gamma (loading control). **b** Western blot detection of ERP29. This example compares Mr/pI from 2D gel and Western blot where the mouse anti-ERP29 detects protein spot #1525, while the rabbit anti-ERP29 detects an additional protein spot, possibly an isoform of ERP29

Confirmation of the protein identification was performed by western blot analysis of the samples used for 2D electrophoresis. Antibodies against 16 proteins were analysed. Protein identities with the highest VIP < 20 were prioritised for analysis. Antibodies were selected based on the qualitative and quantitative recognition of protein bands, and protein detection was confirmed using positive control cell lines (Additional file 1: Table S2). Initially, we analysed several antibodies against each protein and identified useful antibodies for detection of biomarkers from the 2D gels (Fig. 2).

Several isoforms of the identified proteins were detected by western blot. ERP29 was detected in both 2D and western blot at around 29 kDa as a double band, however only the upper band corresponded to protein spot #1525 from the 2D analysis. The identified isoform, with NCBI accession number NP-006808, had both experimental and theoretical pI of around 6.5. The particular band could be singled out by a mouse monoclonal antibody (83,073, Abcam, Cambridge, UK) and was confirmed by 2D Western blot (Fig. 2b). CRKL was located at 25 kDa on 2D and in western blot the correct isoform was identified among four detected bands. Finally, both the 60 kDa and 53 kDa isoforms of SRC were recognised as part of the biomarker panel.

### Verification of platelet biomarkers by western blot and DigiWest data

Expression of proteins in western blot was normalized against the levels of 14–3-3-gamma, which is a more stable and relevant loading control for platelet proteins compared to GAPDH [18]. PCA was performed individually in each set of blotted membranes in order to look for major trends and grouping of the samples by observing the scores plots. These represented three repetitions of the experiment, with four groups per experiment. The PCA scores plots showed consistent separation between benign adnexal lesions from the cases of ovarian cancer, FIGO stages III-IV, as it was observed in 10 of the 12 PCA’s scores plots.

**Fig. 3:**
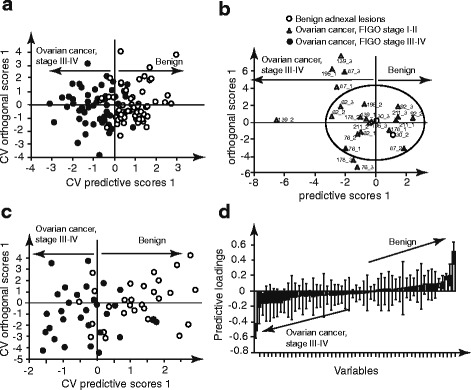
Statistical analysis of the protein expression levels. **a** Cross-validated model built upon protein expression in benign and ovarian cancer, FIGO stage III-IV – western blot data, **b** Test model detecting the cases of ovarian cancer, FIGO stage I-II – western blot data, **c** Cross-validated model built upon protein expression in benign and of ovarian cancer, FIGO stage III-IV – DigiWest data, **d** Relative contribution of variables within the model on the separation between benign adnexal lesions and ovarian cancer, FIGO stage III-IV – DigiWest data

There was nonetheless some intrinsic batch variability, which could potentially be explained primarily by differences in the gels prepared for each batch, uneven protein transfer from gel to membrane, and suboptimal quantification of the protein bands expressed. Due to such variability, data were centred per batch previous to OPLS-DA modeling. After proper validation of the OPLS-DA model as described in the statistical section, results of the verification by western blot revealed a sensitivity of 83% (50/60) and a specificity of 89% (53/60) for separation between benign adnexal lesions and ovarian cancer, FIGO stages III-IV (Fig. 3a, Table 2b).

Next, model was tested for its predictive capacity. The correct prediction of two out of three samples per case was set as an acceptance criterion. Seven out of eight cases of ovarian cancer, FIGO stages I-II were predicted correctly (88% sensitivity). Sample 30 was reclassified by histology as benign and the prediction model confirmed this reclassification (Fig. 3b). Nine protein variables were suggested as a biomarker panel. In ovarian cancer, FIGO stages III-IV, seven protein variables were up-regulated and two variables were down-regulated. The ROC analysis of the detected nine-variable panel suggested discrimination between benign adnexal lesions and ovarian cancer, FIGO stages III-IV with a sensitivity of 60% and a specificity of 83% (Table 2c – ROC1) and between benign adnexal lesions and ovarian cancer, FIGO stages I-II with a sensitivity of 83% and a specificity of 76% (Table 2c – ROC2).

Next, the panel was verified by DigiWest with inclusion of a new, larger set of samples that were not previously used for 2D gel electrophoresis and western blot analysis. The expression profiles were analysed by OPLS-DA and the DigiWest based prediction model suggested a sensitivity of 70% (21/30) and a specificity of 83% (24/29) for the separation between benign adnexal lesions and ovarian cancer, FIGO stages III-IV (Fig. 3c, Table 2d). The verification of biomarker candidates by DigiWest suggested seven protein variables as seen in the loadings plot in Fig. 3d; three protein variables were increased in ovarian cancer, FIGO stages III-IV and four were decreased. We observed a correspondence between western blot and DigiWest analysis regarding the potential value of six identified proteins as biomarkers, of which three were increased in ovarian cancer, FIGO stages III-IV and three were decreased in both western blot and DigiWest.

## Discussion

In this study, we analysed the proteome of platelets isolated from peripheral blood of patients with ovarian cancer, FIGO stages I-II and III-IV, and benign adnexal lesions. By PLS-DA modelling of protein expression in 2D gels we observed the possibility to correctly differentiate between cases of benign adnexal lesions and ovarian cancer, FIGO stages III-IV, and correctly predict cases of FIGO stages I-II. These findings were verified by western blot and DigiWest.

In the prospectively collected clinical material, there were 57 cases of benign adnexal lesions, 49 cases of ovarian cancer, FIGO stages III-IV, and 8 cases of ovarian cancer, FIGO stages I-II. Overrepresentation of advanced ovarian cancer patients (80% of all cases) in our study reflects the worldwide situation regarding the detection rate of the disease at its advanced stages [1]. The initial PCA provided an indication of degree of correspondence between protein profiles of the compared groups. PLS-DA allowed us to build a multivariate regression model. Through PLS-DA it became possible to 1) “filter” the high level of biological background noise by using only the latent variables related to the biological variation of interest, 2) build a training model and use it to predict the class of earlier cancer test samples, and 3) identify the most discriminant variables within the multiplex profile by observing their variable importance in projection (VIP), i.e. ranking in the model [22–24].

Our proteome analysis was based on 2D gel electrophoresis, which made it possible to identify full-length proteins and also their isoforms with regards to their relevance for the protein marker panel. The verification of identified platelet proteins suggested the possibility for separating benign adnexal lesions, ovarian cancer, FIGO stages I-II and III-IV. However, verification by western blot has inherently several disadvantages, such as gel batch variability, protein transfer limitations, and semi-quantitive output. DigiWest, a recently developed system that represents a high-throughput multiplex version of the classical western blot method, uses a bead-based microarray platform for signal generation [19]. The sensitivity, signal linearity, and reproducibility of DigiWest are comparable to or exceed the best available western blot systems. Compared to the western blot method, DigiWest provides higher reproducibility with simultaneous analysis of up to 100 samples and quantification of individual cases.

The function of the 16 identified and immuno-validated platelet proteins was described using the Platelet Web database [25] (Additional file 1: Table S3). Most of these proteins are reported to have a functional relevance to ovarian cancer, normal platelet biology and platelet-associated pathological conditions (Table 4). Published proteomics and molecular biology studies suggest up-regulation of ACTN4, CRKL, GELS, HSPA8, talin-1, tubulins and WDR1 in ovarian cancer. Expression changes and possible functional impact of ACTN1 [26], MPST [27], ERP29 [28] and SERPINB6 [29] are reported for several cancers, but not for ovarian cancer. PHB, SRC, talin-1, tubulins and WDR1 are related to development of ovarian cancer resistance to paclitaxel [30, 31] and cis-platin [32–34]. An interesting observation is an increased expression level of CD41/CD61 in platelets associated with tumor xenografts as well as enhanced tumor growth in the presence of platelets [35].

**Table 4:** Function of proteins biomarkers in relation to ovarian cancer and platelets

Protein name	Associated pathologic conditions	
	ovarian cancer	platelet disorders
ACTN1	not studied	macrotrombocytopenia [37, 38]
ACTN4	↑ in ovarian cancer [39]	myelodysplastic syndrome [38, 40]
CD41/CD61	platelets contribute to ovarian cancer growth [35]	deficient in Glanzmann thrombasthenia type II [41]
CRKL	↑ in ovarian cancer [42]	ST - elevated myocadial infarction [43]
ERP29	not studied	mediator of thrombus formation [44]
GELS	↑ in serum in ovarian cancer [45]	↑ in megakaryoblastic leukemia [46], ↑ in thrombin-activated platelets [47]
HSPA8	↑ in ovarian cancer, a potential therapy target [48]	mediator of thromboembolism [49]
MPST	not studied	not clarified
PHB	↑ in paclitaxel-resistant ovarian cancer [30] ↓ in platinum-resistant ovarian cancer [32]	mediator of platelet aggregation [50]
SRC	therapy-target for thyrosine-kinase inhibitor in ovarian cancer [51], resistance to cis-platin [33]	ST - elevated myocadial infarction [43]
SERPINB6	not studied	inhibitor of thrombin [25]
TLN1	↑ in ovarian cancer [52], resistance to cis-platin [33]	myelodysplastic syndrome [40]
TUBB1, TUBA4	↑ in ovarian cancer, mediator of paclitaxel resistance [31]	↑ in thrombin-activated platelets [47], gene mutation in autosomal dominant macrothrombocytopenia [25]
WDR1	↑ in ovarian cancer [53], resistance to cis-platin [34]	mediator of TLN1-induced activation of CD41/CD61 [54]

↑ corresponds up-regulation, ↓ corresponds down-regulation

**Fig. 4:**
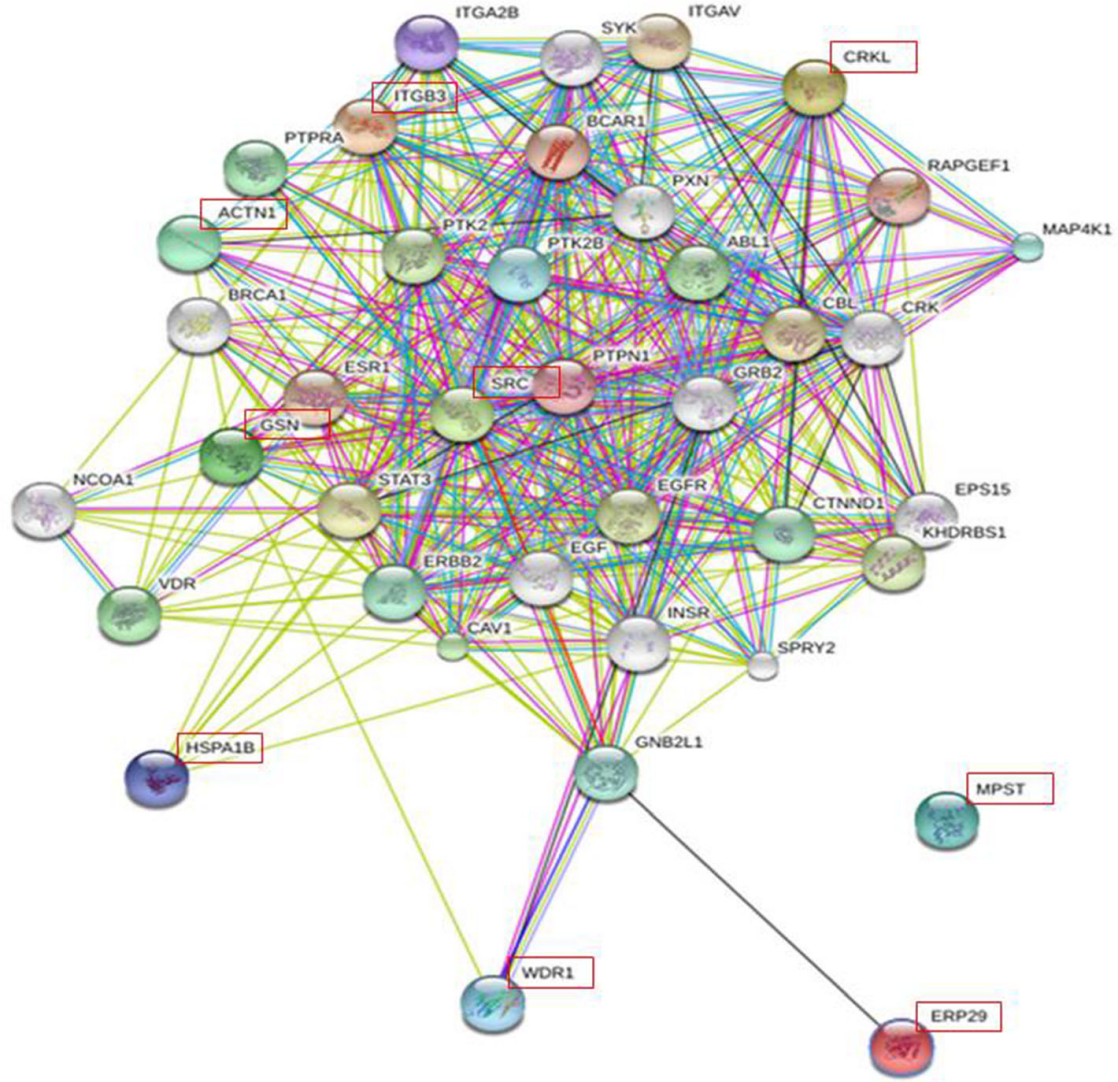
Functional interaction profile of identified platelet proteins [36]. Lines represent interaction, where thick lines suggest a substantial number of references, thin lines correspond to single studies, arrows point the direction of activation influence, and block signs describe inhibitory actions

Using the STRING database [36], we analyzed potential interactions and functional impact of the identified platelet proteins on other known protein targets (Additional file 1: Table S4, Fig. 4). Most described protein interactions are related to CRKL, SRC, CD61, GELS (GSN), and ACTN1, while information is limited regarding the interaction profile of MPST, ERP29, HSPA8, and WDR1.

Limitations of the current study include dependency of sensitivity and specificity on the quantitative properties of the verification method and analysis of relatively small groups of clinical material. Multivariate PLS-DA-based statistics partially allowed us to overcome the problem of small clinical groups, however, further validation using clinical material from patients with early-stage ovarian cancer and borderline ovarian tumors is expected to strengthen the diagnostic sensitivity and specificity. Combination of the marker panel with the TVS protocol, which evaluates malignant potential of adnexal masses, towards a biochemical-instrumental test is the final goal. The objective diagnosis of adnexal lesions will help clinicians to correctly refer patients to a gynaecological cancer specialist, thus minimizing the cost of additional examinations and delays for patients needing advanced treatment. The panel detected is currently being developed as a new non-invasive in vitro diagnostic multivariate index-based analytical method for differentiation of adnexal lesions.

## Conclusion

The identified platelet protein panel allows for differentiation between benign adnexal lesions and ovarian cancers of FIGO stages III-IV. Correct prediction of seven out of eight cases of ovarian cancer, FIGO stages I-II was possible through multivariate prediction modeling based on platelet protein expression profiles.

## Additional file

Additional file 1: Table S1. Detailed description of the clinical material. **Table S2.** Description of the primary antibodies. **Table S3.** Function of proteins biomarkers, relation to ovarian cancer and platelet function. **Table S4.** Predicted functional partners for identified biomarkers. (XLSX 32 kb)

## Abbreviations

2D: Two-dimensional
ANOVA: Analysis of variance
AUC: Area under the curve
CV: Cross validation
DA: Discriminant analysis
FIGO: International Federation of Gynaecology and Obstetrics
IOTA: International Ovarian Tumor Association.
LDS: Lithium dodecyl sulfate.
PCA: Principal components analysis.
PF-4: Platelet factor 4.
PLS: Projection to latent structures (or partial least squares).
PMF: Peptide mass fingerprint.
RCF: Relative centrifugal force.
ROC: Receiver-operator curve.
RMI: Risk of malignancy index.
STED: Stimulated emission depletion.
TVS: Transvaginal ultrasound.
VEGF: Vascular endothelial growth factor.
VIP: Variable importance in projection.
X: Data matrix (number of rows equal to number of samples, number of columns equal to number of variables).
Y: Column vector (with one element per sample).
